# Ivabradine Ameliorates Cardiac Diastolic Dysfunction in Diabetic Mice Independent of Heart Rate Reduction

**DOI:** 10.3389/fphar.2021.696635

**Published:** 2021-06-22

**Authors:** Hao Xie, Xing-Yi Shen, Na Zhao, Peng Ye, Zhen Ge, Zuo-Ying Hu

**Affiliations:** Department of Cardiology, Nanjing First Hospital, Nanjing Medical University, Nanjing, China

**Keywords:** ivabradine, c-Jun N-terminal kinase, p38 mitogen-activated protein kinase, cardiac fibrosis, cardiac diastolic dysfunction

## Abstract

Cardiac fibroblast (CF) proliferation and activation play important roles in cardiac fibrosis and diastolic dysfunction (DD), which are involved in fibrosis-associated cardiovascular diseases. A previous study showed that ivabradine, a specific heart rate (HR)-lowering agent, significantly ameliorated DD in diabetic db/db mice by reducing HR. Herein, we attempted to determine whether ivabradine has antifibrotic and cardioprotective effects in diabetic mice by directly suppressing CF proliferation and activation, independent of a reduction in HR. We found that knockdown of c-Jun N-terminal kinase (JNK) or p38 mitogen-activated protein kinase (MAPK), or treatment with ivabradine, reduced JNK and p38 MAPK phosphorylation and the protein expression of proliferating cell nuclear antigen, collagen I, collagen III, tissue inhibitor of matrix metalloproteinase 2, and α-smooth muscle actin, accompanied with upregulation of matrix metalloproteinase 2 both in high glucose-treated neonatal rat CFs and left ventricular CFs isolated from db/db mice. However, zatebradine (a HR-lowering agent) did not have these effects *in vitro* or *in vivo.* In addition, cardiac fibrosis and DD were ameliorated in db/db mice that were intravenously administered lentiviruses carrying short hairpin RNAs targeting JNK and p38 MAPK or administered ivabradine. Taken together, these findings demonstrate that the ivabradine-induced amelioration of cardiac fibrosis, and DD in db/db mice may be at least in part attributable to the suppression of CF proliferation and activation, through the inhibition of JNK and p38 MAPK.

## Introduction

Diastolic dysfunction (DD) has been identified in many cardiovascular diseases and is associated with poor outcomes, including mortality and hospitalization, owing to heart failure (HF) (Nagueh, 2020). The pathophysiology of DD is multifactorial (Czuriga et al., 2012). A great deal of evidence suggests that cardiac fibrosis, which is characterized by the accumulation of extracellular matrix (ECM) proteins in the cardiac interstitium, is a critical factor in the pathogenesis of DD (Czuriga et al., 2012; Kong et al., 2014; Alex et al., 2018; Ha et al., 2020). Previous studies have suggested that cardiac fibroblast (CF) proliferation and activation drive ECM remodeling, leading to ventricular stiffness and delaying left ventricular (LV) relaxation, which causes DD and ultimately exacerbates the symptoms of HF (Kong et al., 2014; Piccoli et al., 2017). Thus, the inhibition of CF proliferation and activation may ameliorate DD and HF.

It has been demonstrated that the c-Jun N-terminal kinase (JNK) and p38 mitogen-activated protein kinase (p38 MAPK) signaling pathways play important roles in CF proliferation and activation, and that the inhibition of JNK or p38 MAPK signaling ameliorates fibrosis (Meyer-Ter-Vehn et al., 2006; Bansal et al., 2017; Du et al., 2020; Yue et al., 2020). Intriguingly, Schulz et al. have reported that JNK and p38 MAPK phosphorylation is higher in rabbits with pacing-induced HF (Schulz et al., 2003). Therefore, heart rate (HR) reduction may reduce the activation of JNK and p38 MAPK, thereby ameliorating cardiac fibrosis and DD, and ultimately improving HF. However, despite numerous studies and the accumulation of evidence during the past few decades, effective treatments for DD-induced HF remain elusive.

Ivabradine selectively inhibits the cardiac pacemaker I_*f*_ current, which is principally created through hyperpolarization-activated cyclic nucleotide-gated (HCN) channels, and has a specific HR-lowering effect (Zuo et al., 2019). A previous study showed that ivabradine significantly ameliorates DD and HF and preserves the ejection fraction (HFpEF) in diabetic db/db mice by reducing HR (Reil et al., 2013). It has been shown that CFs do not express HCN channel proteins (Biel et al., 2009) and play a pivotal role in the pathogenesis of DD (Kong et al., 2014; Piccoli et al., 2017). In addition, ivabradine has been reported to reduce the activation of JNK and p38 MAPK in various animal models; for example, a murine model of chronic viral myocarditis (Li et al., 2016) and a model of diabetic cardiomyopathy (Zuo et al., 2019). Therefore, we hypothesized that ivabradine has a direct antifibrotic effect by reducing JNK and p38 MAPK activity, which ameliorates cardiac fibrosis and DD, independent of a reduction in HR.

## Materials and Methods

### Reagents

Ivabradine hydrochloride (#HY-B0162A) and zatebradine hydrochloride (#HY-13422) were purchased from MedChem Express (Monmouth Junction, NJ, United States). D-Glucose (#G7528) was obtained from Sigma-Aldrich (St. Louis, MO, United States). Antibodies against JNK, p38 MAPK, p-JNK, p-p38 MAPK, proliferating cell nuclear antigen (PCNA), vimentin, tissue inhibitor of matrix metalloproteinase 2 (TIMP2), α-smooth muscle actin (α-SMA), matrix metalloproteinase 2 (MMP2), and β-actin; and horseradish peroxidase-conjugated secondary antibodies were purchased from Cell Signaling Technology (Beverly, MA, United States). Antibodies against collagen I and collagen III were obtained from the Biorbyt Corporation (Orwell Furlong, Cambridge, United Kingdom). A 3-(4,5-dimethylthiazol-2-yl)-2,5-diphenyltetrazolium bromide (MTT) Cell Proliferation and Cytotoxicity Assay Kit was purchased from Beyotime (Jiangsu, China).

### Cell Culture

Primary cultures of neonatal rat ventricular cardiac fibroblasts (NRCFs) were created and cultured as previously described (Zuo et al., 2019). Briefly, Sprague-Dawley rat pups (1–3 days of age) were decapitated and their hearts were quickly excised and rinsed with cold Hank’s balanced salt solution (HBSS). The left ventricles were then minced and digested in Dulbecco’s-modified Eagle’s medium (DMEM, Gibco-BRL, Rockville, MD, United States) containing 0.1% trypsin (Gibco-BRL) and 0.5 g/L collagenase II (Worthington Biochemical Corporation, Lakewood, NJ, United States) 7 times × 6 min. After each digestion, the supernatant was collected and 10% fetal bovine serum (FBS; Gibco-BRL) was added to stop the digestion. Cells were collected by centrifugation at 1,000 × *g* for 10 min, resuspended in DMEM containing 5.5 mmol/L D-glucose, 10% FBS, 100 U/mL penicillin, and 100 μg/mL streptomycin; and incubated in a humidified atmosphere comprising 5% CO_2_ and 95% air at 37°C. After 1 h of incubation, the NRCFs had attached to the dishes. The medium was replaced every 24 h. After 3 days of culture, when the cells had reached 80% confluence, the cultures were switched to serum-free essential medium overnight, before treatment with the indicated agents. The NRCFs used in the experiments were obtained during passages 2–3.

### Small Interfering (si)RNA Transfection

The NRCFs were transfected with JNK siRNA (ON-TARGETplus SMARTpool, L-088154-02-0005, Dharmacon Inc., Lafayette, CO, United States), p38 MAPK siRNA (ON-TARGETplus SMARTpool, L-080059-02-0005, Dharmacon), or Control siRNA (sc-37007, Santa Cruz, CA, United States) using Lipofectamine RNAiMAX reagent (Invitrogen, Carlsbad, CA, United States), according to the manufacturer’s protocol. The final siRNA concentration was 100 nM.

### Cell Proliferation Assay

NRCF proliferation was assessed using an MTT Cell Proliferation and Cytotoxicity Assay Kit (Beyotime) as previously described (Wu et al., 2016).

### Animals

All animal experimental procedures were approved by the Institutional Animal Care and Use Committee of Nanjing Medical University (Nanjing, China). Seven-week-old male C57BL/6J (20–22 g) and C57BL/KsJ-Lepr^db/db^ (db/db; 36–40 g) mice were obtained from the Model Animal Research Center of Nanjing University. Animals were housed under standard conditions under a 12-h light/dark cycle and were provided with distilled water and chow ad libitum. Mice (*n* = 30) were randomly allocated to three groups: C57BL/6J mice (Control), db/db mice (DM) and db/db mice administered ivabradine (Servier, Tianjing, China) at 20 mg/kg body mass/day (DM + Iva). The latter group was administered ivabradine in their drinking water for 4 weeks, as previously described (Reil et al., 2013). To determine whether a reduction in HR has an antifibrotic effect in diabetic mice, db/db mice (*n* = 30) were randomly allocated to three zatebradine (MedChem Express, Monmouth Junction, NJ, United States) treatment groups: a 5 (5 mg Zate), a 10 (10 mg Zate), and a 20 mg/kg body mass/day (20 mg Zate) group. The C57BL/6J mice (*n* = 30) were also randomly allocated to three groups: a C57BL/6J mouse group (Control), a C57BL/6J mouse group administered ivabradine at 20 mg/kg body mass/day (Control + Iva), and a C57BL/6J mouse group administered zatebradine at 10 mg/kg body mass/day (Control + Zate). Zatebradine was administered via the drinking water and the experimental protocol was the same as that for the ivabradine experiment.

### Lentivirus Injection

In these experiments, the JNK and p38 MAPK genes were knocked down using small hairpin RNA (shRNA) delivered by lentiviruses (LVs). LV-JNK shRNA (titer: 2.78×10^9^ TU/mL), LV-p38MAPK shRNA (titer: 3.36×10^9^ TU/mL), and LV-Scrambled shRNA (titer: 5.11×10^9^ TU/mL) were generated by Genechem (Shanghai, China). The sequences for the JNK, p38 MAPK and Scrambled shRNAs were AAG​AGA​TTT​GTT​ATC​CAA​A, CCA​ACA​ATT​CTG​CTC​TGG​TTA, and TTC​TCC​GAA​CGT​GTC​ACG​T, respectively. Seven-week-old male db/db mice (*n* = 40) were randomly allocated to four groups: db/db + normal saline, db/db + LV-Scrambled shRNA, db/db + LV-JNK shRNA, and db/db + LV-p38 MAPK shRNA. In addition, to verify the efficiency of the JNK and p38 MAPK LV-mediated knockdown, age-matched male C57BL/6J mice (*n* = 24) were allocated to four groups: control, control + LV-Scrambled shRNA, control + LV-JNK shRNA, and control + LV-p38 MAPK shRNA. In all the experiments, the tails of the mice were wiped with alcohol and then slowly injected with 20 μL of LVs packaged with JNK, p38 MAPK or Scrambled shRNA using a 0.5-mL insulin syringe. After 4 weeks, the cardiac function of the mice was assessed using echocardiography, and then the mice were killed and their cardiac fibroblasts were isolated for use in subsequent experiments.

### Body Mass, Blood Glucose, and Electrocardiographic Measurements

For all the experimental mice, HR, body mass, and fasting blood glucose concentration were measured weekly. Electrocardiographic measurements were performed as previously described (Karnabi et al., 2011; Zuo et al., 2019).

### Echocardiographic and Hemodynamic Measurements

Echocardiographic indices were evaluated according to previously described methods (Cappetta et al., 2020). Briefly, left ventricular filling pressure was assessed using transmitral pulsed-wave Doppler echocardiography and a four-chamber view. The mitral valve early wave peak (E wave), atrial wave peak (A wave), E/A ratio, isovolumetric relaxation time (IVRT), and E wave deceleration time (E Dec t) were measured. Invasive pressure-volume analysis was performed as previously reported (Cai et al., 2020). The relaxation time constant (Tau) and −dP/dt were measured as indices of diastolic function using LabChart 8 software (ADInstruments, Colorado Springs, CO, United States).

### Adult Mouse Ventricular Cardiac Fibroblast Preparation

Adult male mice were anesthetized with 1% pentobarbital sodium (30 mg/kg intraperitoneal injection) and their hearts were excised and rinsed in HBSS. The ventricles were minced and digested, as previously described (Tian et al., 2015; Alex et al., 2018); then the cells were collected by centrifugation at 1,000 × g for 10 min, resuspended in DMEM containing 5.5 mmol/L D-glucose, 10% FBS, 100 U/mL penicillin, and 100 μg/mL streptomycin; and incubated in a humidified atmosphere comprising 5% CO_2_ and 95% air at 37°C. After 1.5 h of incubation, the cells had attached to the dishes, were washed with HBSS, and were then collected by centrifugation at 1,000 × g for 10 min. These cells were used for identification, lysate preparation, and western blotting.

### Western Blotting

Lysates were prepared from cells and cardiac tissue for western blotting analysis according to previously described methods (Zuo et al., 2019).

### Histological Assessment of Fibrosis

The hearts of mice were isolated, fixed in 4% paraformaldehyde solution, and embedded in paraffin. The left ventricles were cut into 5-µm-thick sections, which were stained with Massonʼs trichrome for the evaluation of fibrosis. The stained sections were viewed using a microscope (original magnification × 400; Nikon, Tokyo, Japan).

### Immunofluorescence and Immunohistochemistry

Cells were immunostained for vimentin to confirm that they were NRCFs and isolated adult male left ventricular fibroblasts. Greater synthesis of ECM proteins, such as collagens, is an indicator of fibroblast activation (Alex et al., 2018). To evaluate cardiac fibroblast activation *in vivo,* the levels of collagen I and collagen III, which constitute 90% of all collagen, were determined immunohistochemically, as previously described (Alex et al., 2018). Immunostained sections were examined using a confocal laser scanning microscope coupled to an image analysis system (original magnification × 400; Leica, Wetzlar, Germany).

### Statistical Analysis

Data are expressed as the mean ± standard error of the mean (SEM). Differences between groups were compared using Student’s *t*-test or one-way or two-way ANOVA in GraphPad Prism software 5.0 (GraphPad Inc., San Diego, CA, United States). When statistical significance was identified using ANOVA, a Tukey multiple comparison test was performed. *p* < 0.05 was considered to represent statistical significance.

## Results

JNK and p38 MAPK mediate high glucose-induced NRCF proliferation and activation and the upregulation of ECM protein expression.

**FIGURE 1:**
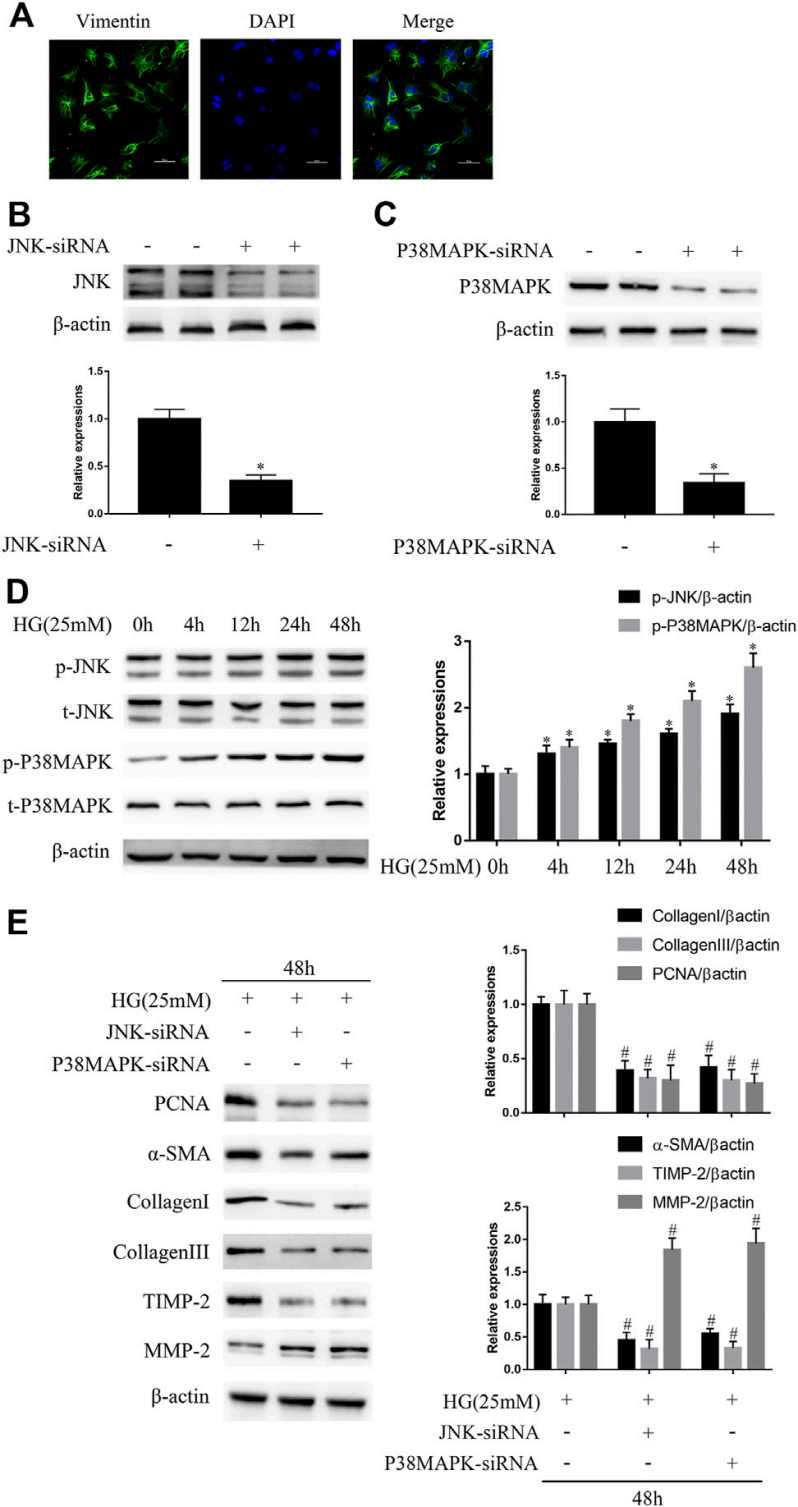
JNK and p38 MAPK mediate high glucose (HG)-induced fibrogenic protein upregulation *in vitro* **(A)** NRCFs were confirmed by vimentin and DAPI staining (original magnification × 400). Scale bars, 50 μm. Data were obtained from three independent experiments **(B and C)** NRCFs transfected with or without JNK or p38 MAPK siRNA were evaluated for the protein expression of JNK and p38 MAPK by western blotting **(D)** NRCFs were exposed to HG for 0–48 h and JNK/p38 MAPK phosphorylation and total JNK/p38 MAPK expression were determined by western blotting **(E)** NRCFs were transfected with or without JNK/p38 MAPK siRNA and then exposed to HG for the indicated periods of time. The protein expression of PCNA, α-SMA, TIMP2, collagen I, collagen III, and MMP2 was determined by western blotting. Data in **(B–E)** are presented as mean ± SEM (*n* = 3). *p*-values in **(B–D)** were calculated using paired Student’s *t*-test. *p*-value in **(E)** was calculated using one-way ANOVA with Tukey multiple comparison test. *p < 0.05, compared to the normal group; ^#^ *p* < 0.05, compared to the HG group.

As shown in Figure 1A, the identity of NRCFs was confirmed by vimentin staining. Next, we confirmed the efficiency of knockdown using siRNAs. Compared with the control, JNK siRNA reduced JNK protein expression by approximately 65.2% and p38 MAPK siRNA reduced p38 MAPK protein expression by approximately 68% (Figures 1B,C). The negative control siRNA had no effect on JNK or p38 MAPK expression (data not shown). JNK and p38 MAPK phosphorylation, but not total JNK and p38 MAPK expression, increased in a time-dependent manner and peaked at 48 h in high glucose (HG)-cultured NRCFs (Figure 1D). Furthermore, after the transfection of JNK or p38 MAPK siRNA, the protein expression of PCNA, α-SMA, TIMP2, collagen I, and collagen III was significantly lower, whereas that of MMP2 was higher in HG-treated NRCFs (Figure 1E). These effects were not observed in NRCF cultures in medium with a normal glucose concentration (Supplementary Figure S1). These data indicate that JNK and p38 MAPK play critical roles in the increase in fibrogenic protein expression that is induced by a high glucose concentration.

JNK or p38 MAPK knockdown attenuates diabetes-induced CF proliferation and activation and reduces ECM protein expression.

**FIGURE 2:**
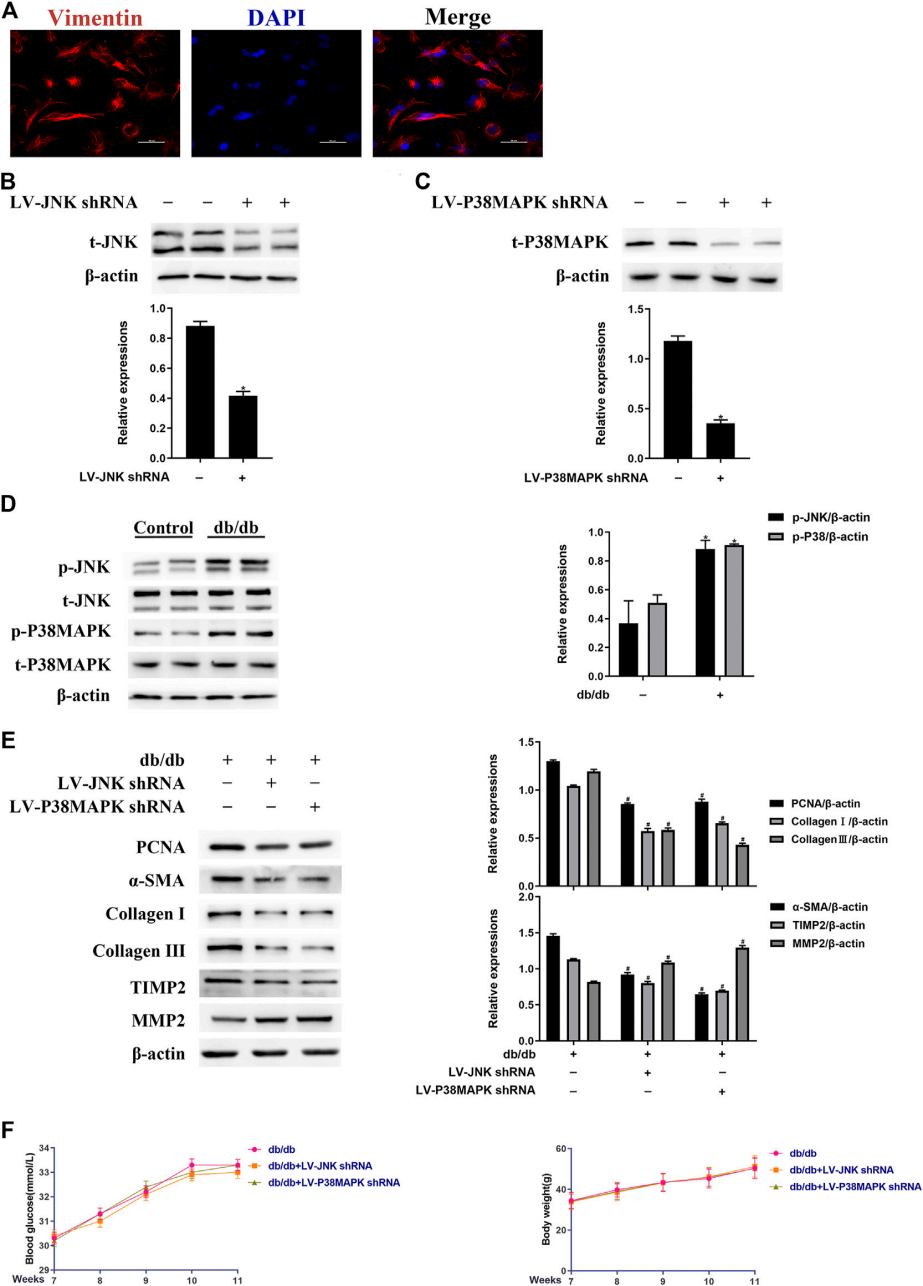
JNK and p38 MAPK mediate the diabetes-induced increase in fibrogenic protein expression *in vivo* **(A)** Left ventricular fibroblasts isolated from mice were confirmed by vimentin staining. Data was obtained from three independent experiments **(B and C)** Isolated left ventricular fibroblasts from wild-type mice that were or were not injected with lentivirus via a tail vein were evaluated for JNK and p38 MAPK protein expression after 4 weeks **(D)** Left ventricular fibroblasts were isolated from mice and JNK and p38 MAPK phosphorylation and total JNK and p38 MAPK expression were determined by western blotting **(E)** Isolated left ventricular fibroblasts from diabetic mice that had or had not been injected with lentivirus via a tail vein were evaluated for PCNA, α-SMA, TIMP2, collagen I, collagen III, and MMP2 expression after 4 weeks **(F)** Between 7 and 11 weeks following the tail vein injection of lentivirus, the blood glucose concentration and body mass of the mice were measured. Data in **(B–F)** are presented as mean ± SEM (*n* = 6). *p*-values in **(B–D)** were calculated using paired Student’s *t*-test. *p*-values in **(E-F)** were calculated using one-way ANOVA with Tukey multiple comparison test. *p < 0.05, compared to the Control group; ^#^ *p* < 0.05, compared to the db/db mice group.

To determine whether JNK and p38 MAPK have profibrotic effects in experimental diabetic cardiomyopathy, LV-mediated knockdown of JNK or p38 MAPK was performed in mice by tail vein injection. The identity of left ventricular fibroblasts isolated from mouse hearts was confirmed by vimentin staining (Figure 2A). As shown in Figures 2B,C, LV-JNK shRNA reduced JNK protein expression by ∼59% and LV-p38 MAPK shRNA reduced p38 MAPK protein expression by ∼63% compared to the control. In contrast, the negative control virus had no inhibitory effect on JNK or p38 MAPK expression (data not shown). Next, we found that while total JNK and p38 MAPK expression was not affected, JNK and p38 MAPK phosphorylation was much higher in left ventricular fibroblasts isolated from db/db mice than in fibroblasts isolated from wild-type mice (Figure 2D). The protein expression of PCNA, α-SMA, TIMP2, collagen I, and collagen III was lower, whereas that of MMP2 was higher in db/db mice administered LV-JNK shRNA or LV-p38 MAPK shRNA than in untreated db/db mice (Figure 2E). However, there were no differences in these parameters in wild-type mice in which JNK or p38MAPK had or had not been knocked down (Supplementary Figure S2A). Finally, compared with untreated db/db mice, knockdown of JNK or p38 MAPK in db/db or wild-type mice did not alter their blood glucose concentration or body mass (Figure 2F; Supplementary Figure S2B). These results imply that JNK and p38 MAPK mediate the diabetes-induced increase in fibrogenic protein expression.

JNK or p38 MAPK knockdown ameliorates diabetes-induced cardiac fibrosis and cardiac dysfunction in diabetic mice.

**FIGURE 3:**
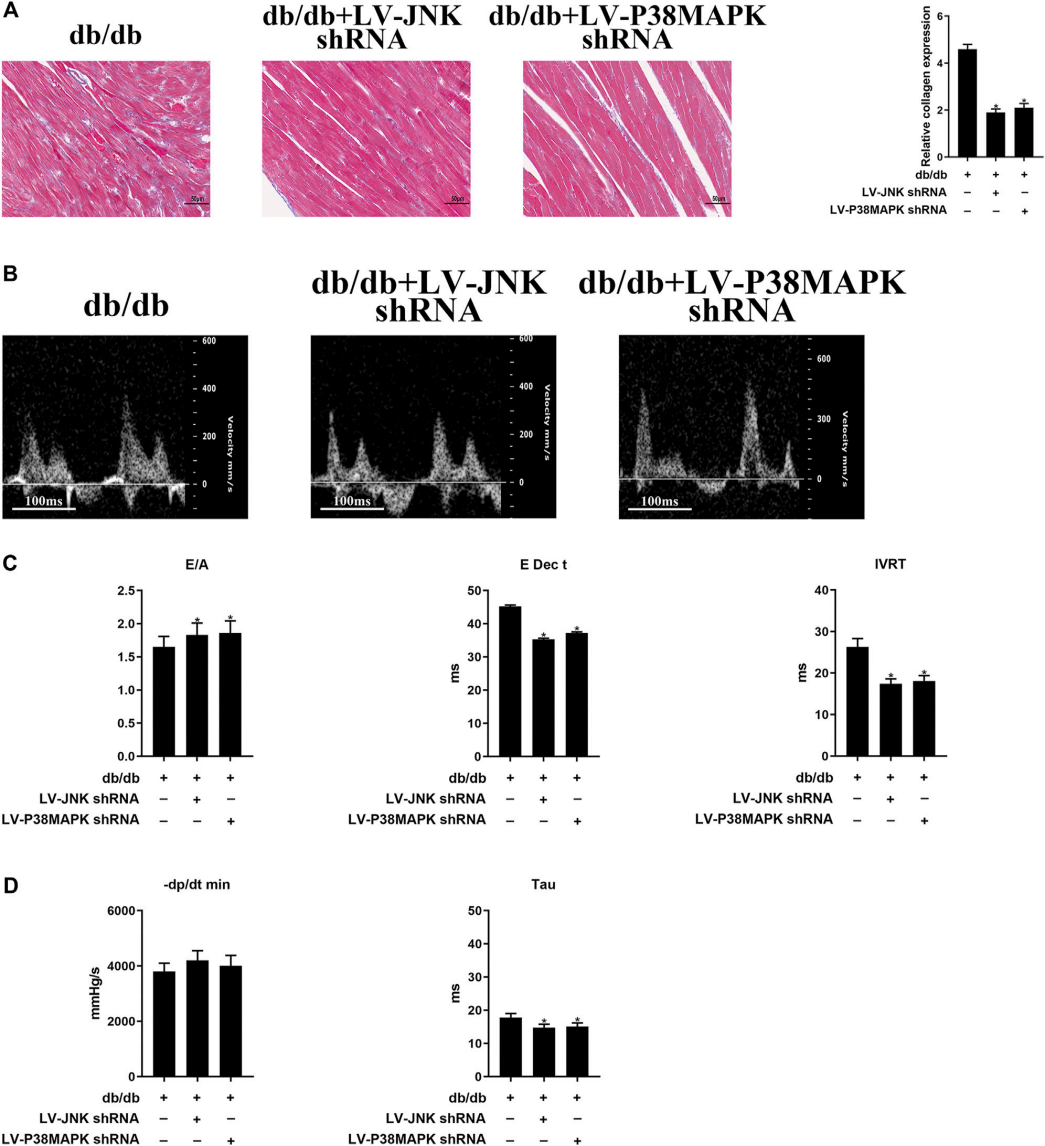
Myocardial fibrosis and cardiac diastolic dysfunction (DD) are ameliorated by the knockdown of JNK or p38 MAPK **(A)** Massonʼs trichrome staining of heart tissue (original magnification ×400). The columns show the differences in collagen accumulation. Scale bars, 50 μm **(B)** Echo-Doppler traces for transmitral flow **(C)** Transmitral pulsed-wave Doppler measurements of E/A, E Dec t, and IVRT **(D)** Hemodynamic parameters: −dP/dt min and the time constant, Tau. The columns show the differences in left ventricular DD and hemodynamic parameters. Data are presented as mean ± SEM (*n* = 6). *p*-values were calculated using one-way ANOVA with Tukey multiple comparison test. *p < 0.05, compared to the db/db mice groups.

As shown in Figure 3A, the area of cardiac fibrosis was smaller in db/db mice administered LV-JNK shRNA or LV-p38 MAPK shRNA than in untreated db/db mice. Furthermore, compared to the untreated db/db mice, JNK/p38 MAPK knockdown in db/db mice significantly ameliorated cardiac DD, as indicated by a high E/A ratio and low E Dec t and IVRT (Figures 3B,C). The beneficial effects of JNK or p38 MAPK knockdown on diastolic performance were also evident in the hemodynamic analysis, with a significant longer Tau (Figure 3D). However, there were no differences in these parameters in wild-type mice in which JNK or p38MAPK had been knocked down or not (Supplementary Figures S3A–D). These results imply that JNK and p38 MAPK play important roles in diabetes-induced cardiac fibrosis and DD.

Ivabradine, but not zatebradine, reduces JNK/p38 MAPK phosphorylation, HG-induced NRCF proliferation and activation, and ECM protein expression.

**FIGURE 4:**
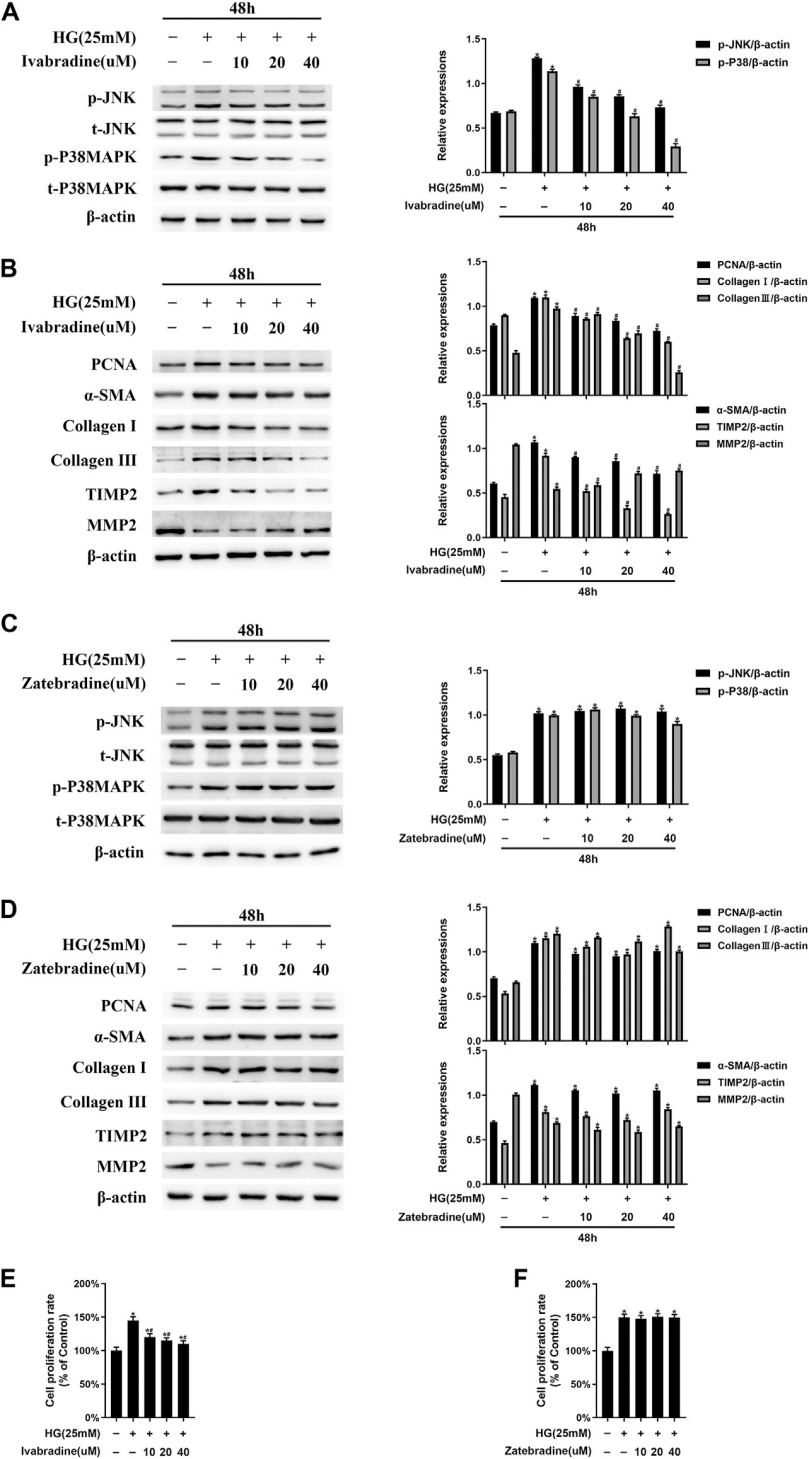
Ivabradine, but not zatebradine, reduces JNK/p38 MAPK activation and the high glucose (HG)-induced increase in fibrogenic protein expression *in vitro* **(A)** NRCFs were pretreated with or without ivabradine at the indicated concentrations for 30 min, and then exposed to HG for 48 h. JNK and p38 MAPK phosphorylation and total JNK and p38 MAPK expression were determined by western blotting **(B)** NRCFs were treated as described in **(A)** and the protein expression of PCNA, α-SMA, TIMP2, collagen I, collagen III, and MMP2 was determined by western blotting **(C and D)** NRCFs were pretreated with or without zatebradine at the indicated concentrations for 30 min, and then exposed to HG for 48 h. The proteins described in **(A)** and **(B)** were quantified by western blotting **(E and F)** NRCFs were treated as described in **(A)** and **(C)** and the cell proliferation rate was determined using an MTT assay. Data are presented as mean ± SEM (*n* = 3). *p*-values were calculated using one-way ANOVA with Tukey multiple comparison test. *p < 0.05, compared to the normal group; ^#^ *p* < 0.05, compared to the HG group.

As shown in Figure 4A, in NRCFs pretreated with ivabradine for 30 min and then exposed to HG for 48 h, JNK/p38 MAPK phosphorylation was markedly inhibited, whereas the total JNK/p38 MAPK expression was not affected. Furthermore, the expression of ECM proteins, such as TIMP2, collagen I, and collagen III, as well as PCNA and α-SMA, was significantly lower in ivabradine-pretreated NRCFs exposed to HG, whereas the protein expression of MMP2 was higher (Figure 4B). Interestingly, zatebradine, an established selective HCN channel inhibitor, did not have similar effects in HG-exposed NRCFs (Figures 4C,D). In addition, ivabradine, but not zatebradine, markedly reduced the proliferation rate of HG-treated NRCFs (Figures 4E,F). These findings demonstrate that ivabradine inactivates JNK and p38 MAPK, and inhibits NRCF proliferation and activation, independent of an inhibitory effect on HCN channels.

Treatment with HR-lowering agents reduces HR, but does not affect the blood glucose concentration or body mass of db/db mice.

**FIGURE 5:**
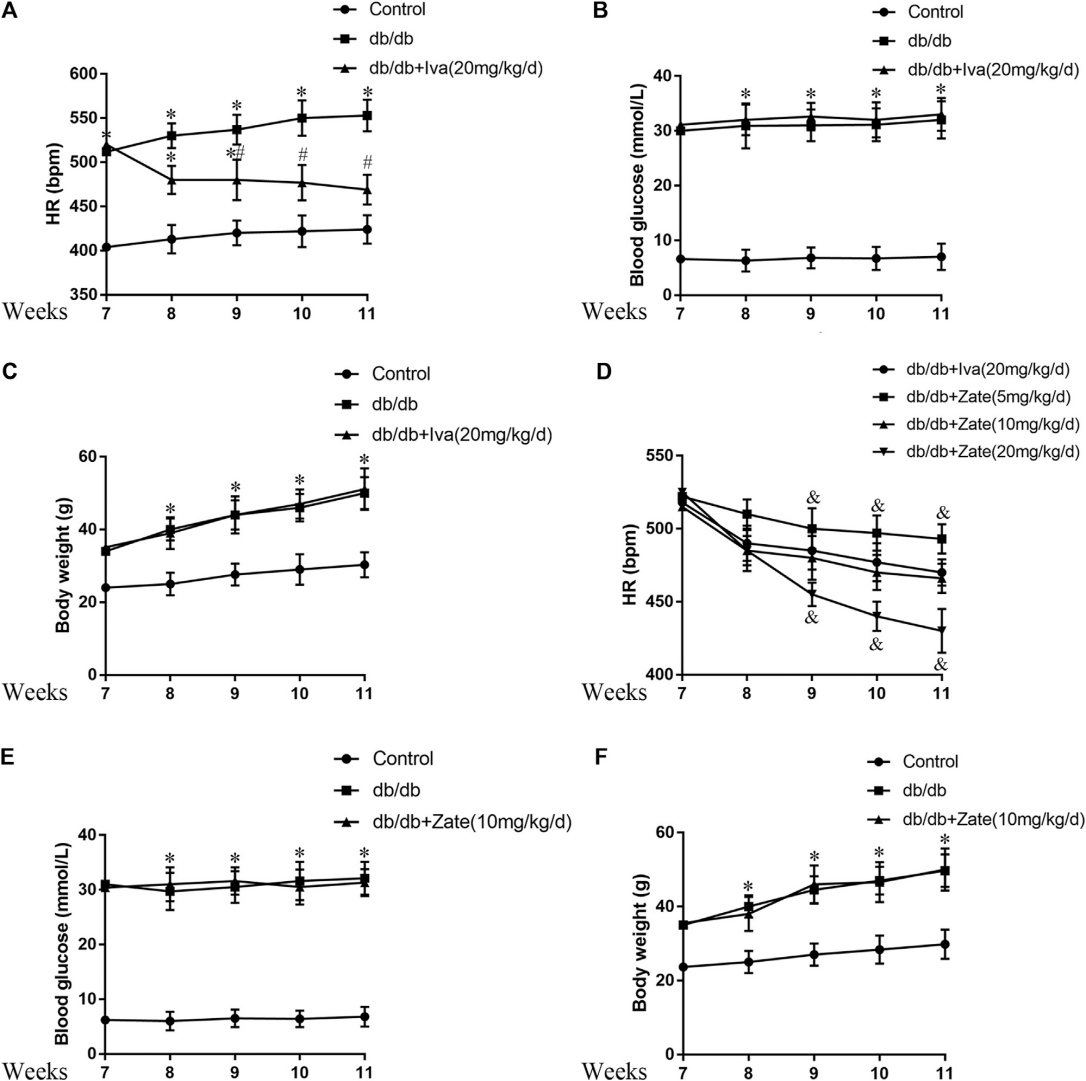
Effects of ivabradine and zatebradine on heart rate (HR) and biometric parameters **(A)** During treatment with or without ivabradine for 7–11 weeks, the mean HR of mice was recorded by electrocardiography (ECG) **(B and C)** Mice were treated as described in **(A)** and their blood glucose concentration and body mass were measured **(D)** During treatment with ivabradine or zatebradine at the indicated doses for 7–11 weeks, the mean HR of mice was recorded by ECG **(E and F)** During treatment with 10 mg/kg/day zatebradine for 7–11 weeks, the blood glucose concentration and body mass of the mice were measured. Data are presented as mean ± SEM (*n* = 8–10). *p*-values were calculated using one-way ANOVA with Tukey multiple comparison test. *p < 0.05, compared to the Control group; ^#^ *p* < 0.05, compared to the db/db mice group; ^&^ *p* < 0.05, compared to the db/db + Iva (20 mg/kg/d) group or the db/db + Zate (10 mg/kg/d) group.

The db/db mice exhibited high HR, blood glucose concentration, and body mass during the experiment. Ivabradine (20 mg/kg/d) administration significantly reduced the HR, but did not have hypoglycemic or weight loss effects (Figures 5A–C). On the basis of previous publications (Stieber et al., 2006; Yamabe et al., 2007), we administered 5, 10, or 20 mg/kg/day zatebradine and determined the effects on the HR of the mice. We found that 10 mg/kg/day zatebradine had the same HR-reducing effect as 20 mg/kg/day ivabradine (Figure 5D). Zatebradine treatment also had no effect on the blood glucose concentration or body mass of db/db mice (Figures 5E,F). Furthermore, although the administration of ivabradine (20 mg/kg/d) or zatebradine (10 mg/kg/d) markedly reduced HR, these drugs did not affect glycemia or body mass (Supplementary Figure S4). These results imply that both ivabradine and zatebradine do not have anti-hyperglycemic or weight loss effects in db/db mice.

Ivabradine reduces the phosphorylation of JNK and p38 MAPK and the proliferation and activation of CFs in db/db mice.

**FIGURE 6:**
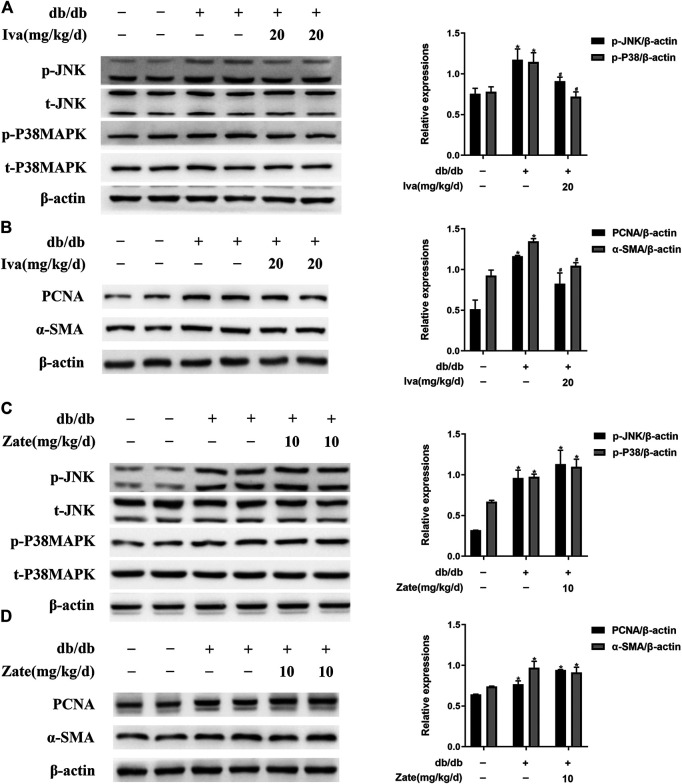
Ivabradine reduces JNK and p38 MAPK activation and inhibits diabetes-induced CF proliferation and activation *in vivo* **(A)** Left ventricular fibroblasts isolated from mice treated with ivabradine at the indicated dose. JNK and p38 MAPK phosphorylation and total JNK and p38 MAPK expression were determined by western blotting **(B)** In CFs from mice treated as described in **(A)**, the expression of PCNA and α-SMA was determined by western blotting **(C and D)** In left ventricular fibroblasts isolated from mice treated with zatebradine at the indicated dose the expression of the proteins listed in **(A)** and **(B)** was measured by western blotting. Data are presented as mean ± SEM (*n* = 3). *p*-values were calculated using one-way ANOVA with Tukey multiple comparison test. *p < 0.05, compared to the Control group; ^#^ *p* < 0.05, compared to the db/db mice group.

As shown in Figure 6A, JNK and p38 MAPK phosphorylation was significantly lower in CFs isolated from the hearts of db/db mice administered ivabradine than in CFs isolated from db/db mice, whereas there was no difference in the total protein expression of JNK or p38 MAPK. In addition, ivabradine administration significantly reduced the expression of PCNA and α-SMA in CFs (Figure 6B). However, zatebradine did not have a similar inhibitory effect on JNK or p38 MAPK phosphorylation or the expression of PCNA or α-SMA in CFs (Figures 6C,D). There were no differences in the expression or phosphorylation of these proteins in wild-type mice administered ivabradine or zatebradine (Supplementary Figure S5). This implies that ivabradine reduces JNK and p38 MAPK phosphorylation and cardiac fibroblast proliferation and activation, independent of a reduction in HR.

Ivabradine reduces ECM protein expression, cardiac fibrosis, and cardiac DD in db/db mice.

**FIGURE 7:**
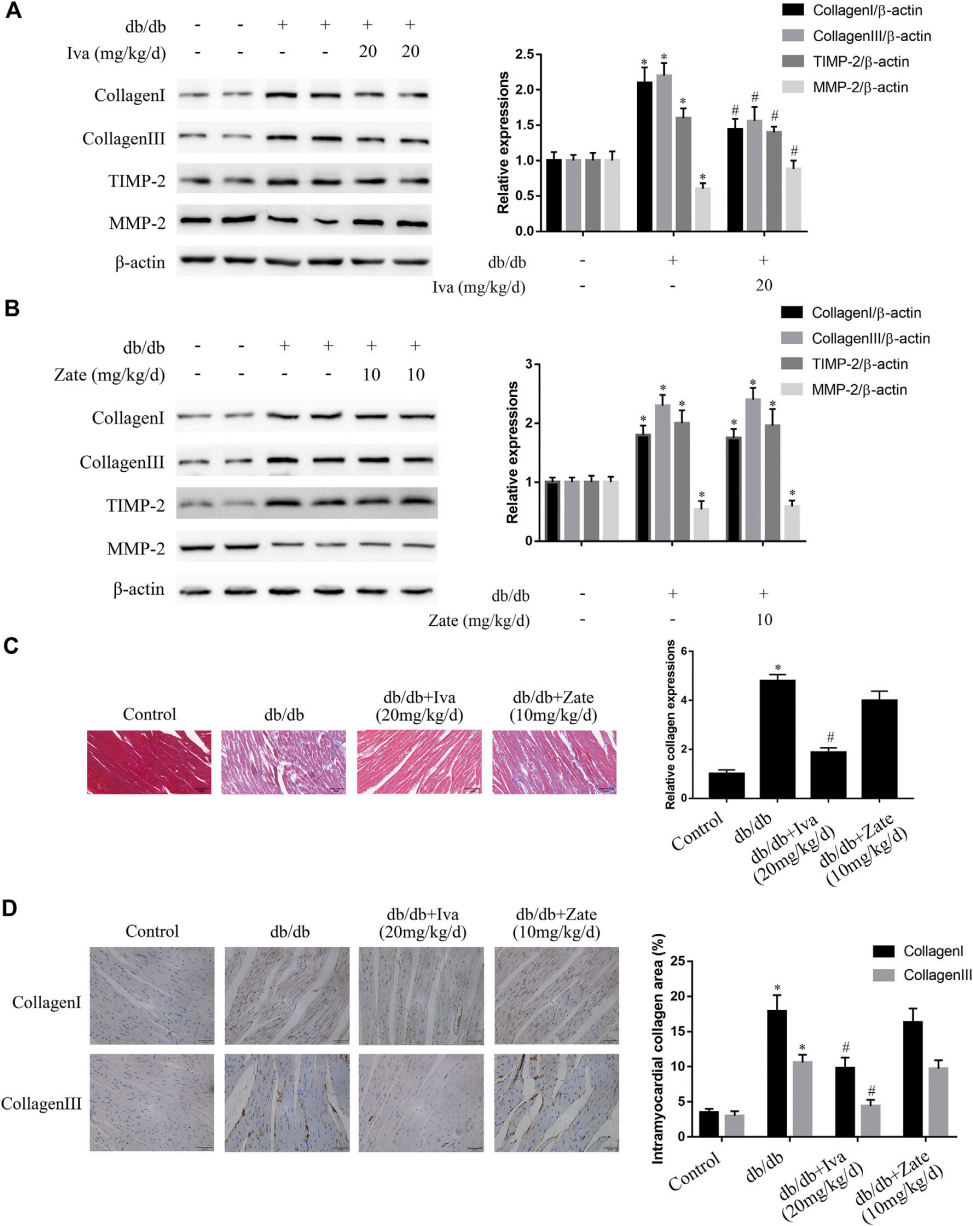
Ivabradine, but not zatebradine, ameliorates the diabetes-induced increases in fibrogenic protein expression and fibrosis **(A)** Collagen I, collagen III, TIMP2, and MMP2 protein expression was determined by western blotting in left ventricular fibroblasts isolated from mice administered ivabradine at the indicated doses **(B)** Left ventricular fibroblasts were isolated from mice administered zatebradine at the indicated doses and the expression of the proteins listed in **(A)** was measured by western blotting **(C and D)** Mice were treated as described in **(A)** and **(B)** and the degree of cardiac fibrosis was determined by Massonʼs trichrome staining and immunohistochemistry. The columns show the differences in collagen accumulation (original magnification ×400). Scale bars, 50 μm. Data in **(A–B)** (*n* = 3) and **(C–D)** (*n* = 6) are presented as mean ± SEM. *p*-values were calculated using one-way ANOVA with Tukey multiple comparison test. *p < 0.05, compared to the Control group; ^#^ *p* < 0.05, compared to the db/db mouse group.

**FIGURE 8:**
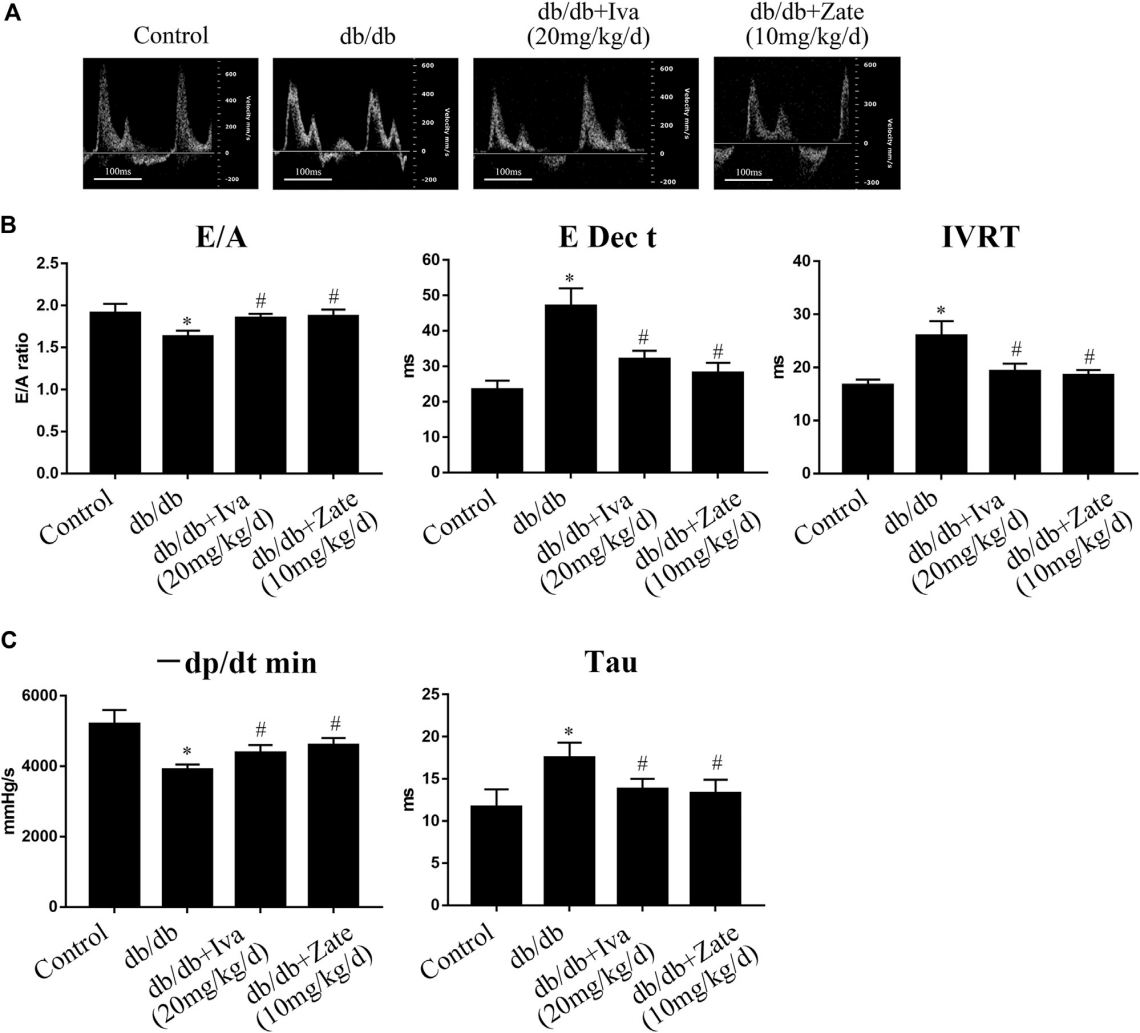
Ivabradine and zatebradine ameliorate diabetes-induced cardiac diastolic dysfunction (DD) **(A)** Mice were administered ivabradine or zatebradine at the indicated doses and echo-Doppler traces for the transmitral flow were recorded **(B)** Mice were treated as described in **(A)** and transmitral pulsed-wave Doppler measurements of E/A, E Dec t, and IVRT were obtained **(C)** Mice were treated as described in **(A)** and the hemodynamic parameters −dP/dt min and Tau were measured. The columns show the differences in left ventricular DD and hemodynamic parameters. Data are presented as mean ± SEM (*n* = 6). *p*-values were calculated using one-way ANOVA with Tukey multiple comparison test. *p < 0.05, compared to the Control group; ^#^ *p* < 0.05, compared to the db/db mouse group.

As shown in Figure 7A, in left ventricular fibroblasts isolated from db/db mice administered ivabradine, the protein expression of collagen I, collagen III, and TIMP2 was significantly lower, while that of MMP2 was higher than in fibroblasts isolated from untreated db/db mice. However, these effects were not observed in fibroblasts isolated from db/db mice administered zatebradine (Figure 7B). Furthermore, ivabradine significantly ameliorated cardiac fibrosis in db/db mice, whereas zatebradine did not (Figures 7C,D). However, there were no differences in fibrogenic protein expression or fibrosis in wild-type mice administered ivabradine or zatebradine or controls (Supplementary Figure S6). Compared to untreated db/db mice, the administration of ivabradine or zatebradine to db/db mice significantly improved cardiac DD, as indicated by a higher E/A ratio and shorter E Dec t and IVRT (Figures 8A,B). The beneficial effects of treatment with ivabradine or zatebradine on diastolic performance were also evident with respect to hemodynamics (Figure 8C). These findings imply that ivabradine has antifibrotic effects in diabetic mice, independent of a reduction in HR.

## Discussion

A body of evidence shows that DD plays a major role in HF and determines prognosis (Burlew and Weber, 2002; Ha et al., 2020; Nagueh, 2020). Histological studies of heart tissue from patients with DD abnormalities have demonstrated greater collagen deposition (Beltrami et al., 1994), which is indicative of cardiac fibrosis. Fibrosis aggravates diastolic suction and passive stiffness, leading to increases in the atrial and ventricular filling pressures, and is considered to be an important cause of DD (Burlew and Weber, 2002). Therefore, investigations of the underlying mechanism of cardiac fibrosis and of potential antifibrotic agents have significant clinical value. Previous studies have suggested that CF proliferation and activation worsen cardiac fibrosis, which leads to ventricular stiffness and delayed left ventricular relaxation, causing DD and ultimately exacerbating the symptoms of HF (Kong et al., 2014; Piccoli et al., 2017). Therefore, the inhibition of CF proliferation and activation may ameliorate DD and HF.

It has been widely reported that JNK and p38 MAPK participate in the proliferation and activation of CFs (Meyer-Ter-Vehn et al., 2006; Bansal et al., 2017; Du et al., 2020; Yue et al., 2020). In the present study, we found high levels of JNK and p38 MAPK phosphorylation in HG-treated CFs and CFs from diabetic mice. In addition, an amelioration of cardiac fibrosis and lower PCNA and α-SMA protein expression were identified following the knockdown of JNK or p38 MAPK, both *in vitro* and *in vivo,* and these findings are consistent with those of previous studies (Meyer-Ter-Vehn et al., 2006; Bansal et al., 2017; Du et al., 2020; Yue et al., 2020). Schulz et al. found that JNK and p38 MAPK phosphorylation is higher in rabbits with pacing-induced HF (Schulz et al., 2003). Furthermore, intriguingly, Zuo et al. showed that type I diabetic mice have a high HR (Zuo et al., 2019), and Hillis et al. reported that patients with type 2 diabetes mellitus, who have a higher resting HR, are at higher risk of death and cardiovascular complications (Hillis et al., 2012). Therefore, methods that reduce HR may be able to reduce the HG and diabetes-induced phosphorylation of JNK and p38 MAPK and the subsequent CF proliferation and activation. In the present study, in HG-cultured NRCFs, pretreatment with ivabradine, but not zatebradine, inhibited JNK and p38 MAPK phosphorylation and CF proliferation and activation. Furthermore, in diabetic mice, the administration of ivabradine or zatebradine significantly reduced HR, but had no effect on blood glucose concentration or body mass. However, after 11 weeks of administration of ivabradine, lower JNK and p38 MAPK activation and antifibrotic effects were identified in left ventricular fibroblasts isolated from db/db mice, whereas zatebradine did not have these effects. The discrepancy in the effects of these two HR-lowering agents on CFs subjected to a HG environment requires further investigation, but the underlying mechanism of the antifibrotic effect of ivabradine may be of significant clinical value.

The pacemaker current (I_*f*_) of sinoatrial cells, which is derived from HCN channels, determines the rapidity of diastolic depolarization, thereby controlling HR (Herrmann et al., 2007; Dilaveris et al., 2006). Ivabradine and zatebradine have been shown in many previous studies to be HCN channel inhibitors (Li et al., 2016; Dunlop et al., 2009). In addition, Kleinbongard et al. found that ivabradine has pleiotropic cardioprotective effects that are independent of a reduction in HR, and also has direct anti-ischemic effects *in vitro* (Kleinbongard et al., 2015). In the present study, both ivabradine and zatebradine markedly ameliorated diabetes-induced cardiac DD. However, only ivabradine had an inhibitory effect on the phosphorylation of JNK and p38 MAPK and the proliferation and activation of HG-treated NRCFs and CFs isolated from the hearts of diabetic mice. Because HCN channels are not expressed in CFs (Li et al., 2016), we propose that ivabradine has its antifibrotic effects in a manner that is independent of HCN channel inhibition, and therefore a reduction in HR. However, whether ivabradine reduces the phosphorylation of JNK and p38 MAPK directly or via other molecules should be determined in further research.

Taken together, the present findings demonstrate that the ivabradine-induced amelioration of cardiac DD in db/db mice may be at least in part attributable to reductions in the proliferation and activation of CFs, through the inhibition of JNK and p38 MAPK signaling. These findings suggest that ivabradine may represent a promising antifibrotic drug for the treatment of fibrosis-associated cardiovascular diseases, such as obesity-related fibrotic cardiomyopathy and HF with preserved ejection fraction (Alex et al., 2018).

### Study Limitations

Transforming growth factor beta (TGF-β) binds to a heterodimer of the TGF-β type I (TβRI) and TβRII receptors, which causes the phosphorylation of TGF-β-activated kinase 1 (TAK1) and the activation of downstream JNK and p38 MAPK signaling, independent of the “suppressor of mothers against decapentaplegic” (SMAD) pathway. This initiates the activation of CFs and the progression of fibrogenesis (Bansal et al., 2017; Khalil et al., 2017). In diabetes, hyperglycemia activates TGF-β, which leads to cardiac fibrosis, DD, and diabetic cardiomyopathy, which involves JNK and p38 MAPK signaling (Yue et al., 2017). In addition, ivabradine treatment significantly reduces TGF-β protein expression in myocardial infarction (Dedkov et al., 2007). We speculate that ivabradine inhibits TGF-β protein expression and that of downstream molecules, such as TβRI, TβRII, and TAK1, leading to lower activation of JNK and p38 MAPK in diabetic hearts. Although we did not determine whether ivabradine reduces TGF-β expression and downstream signaling in the present study, these questions will be addressed in a future study.

## Data Availability

The original contributions presented in the study are included in the article/Supplementary Material, further inquiries can be directed to the corresponding authors.
