# Supplementary figures and images for: Ivabradine Ameliorates Cardiac Diastolic Dysfunction in Diabetic Mice Independent of Heart Rate Reduction
Source: Front Pharmacol. 2021 Jun 22;12:696635. doi: 10.3389/fphar.2021.696635 (PMC8259788; doi:10.3389/fphar.2021.696635)

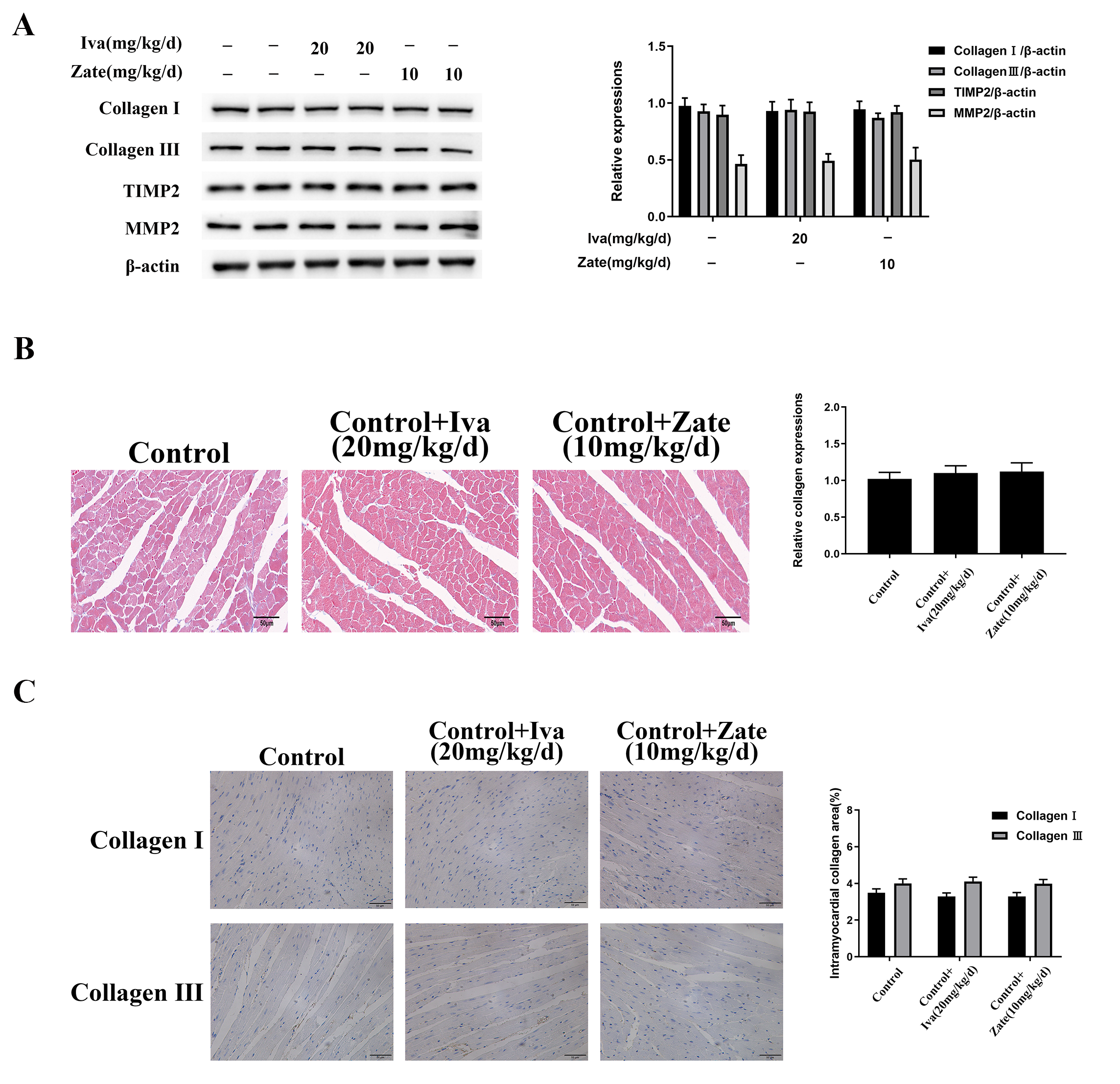

Supplement: Supplementary file 1 [file Image6.TIF]

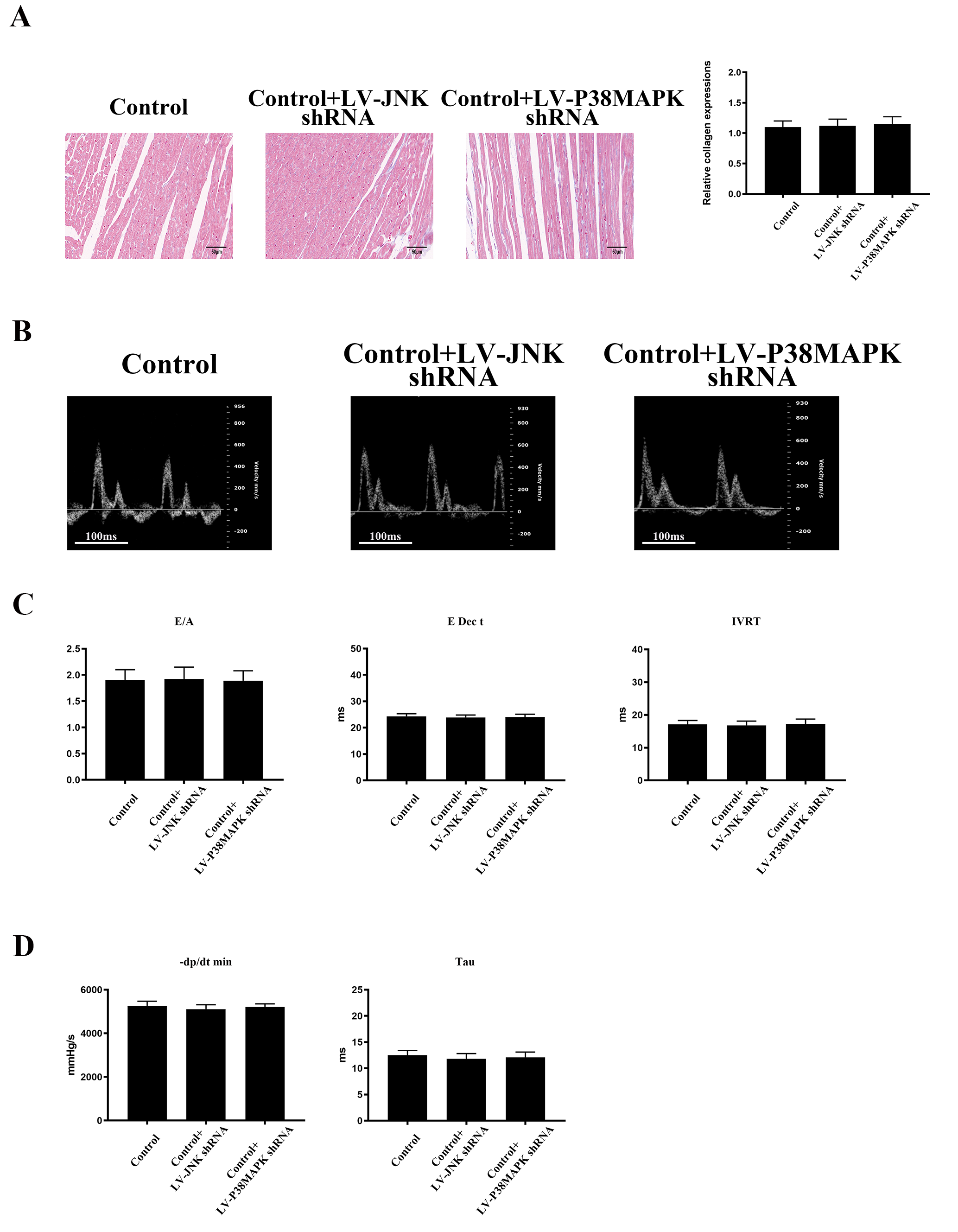

Supplement: Supplementary file 2 [file Image3.TIF]

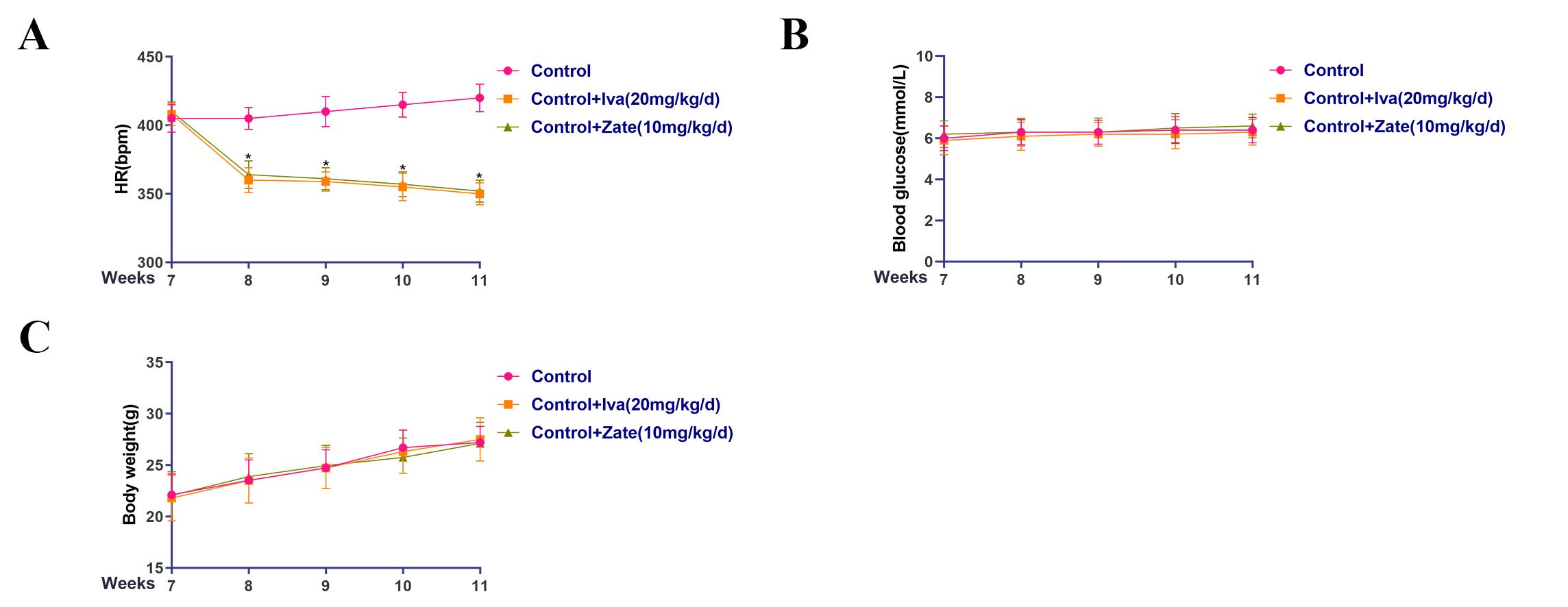

Supplement: Supplementary file 3 [file Image4.TIF]

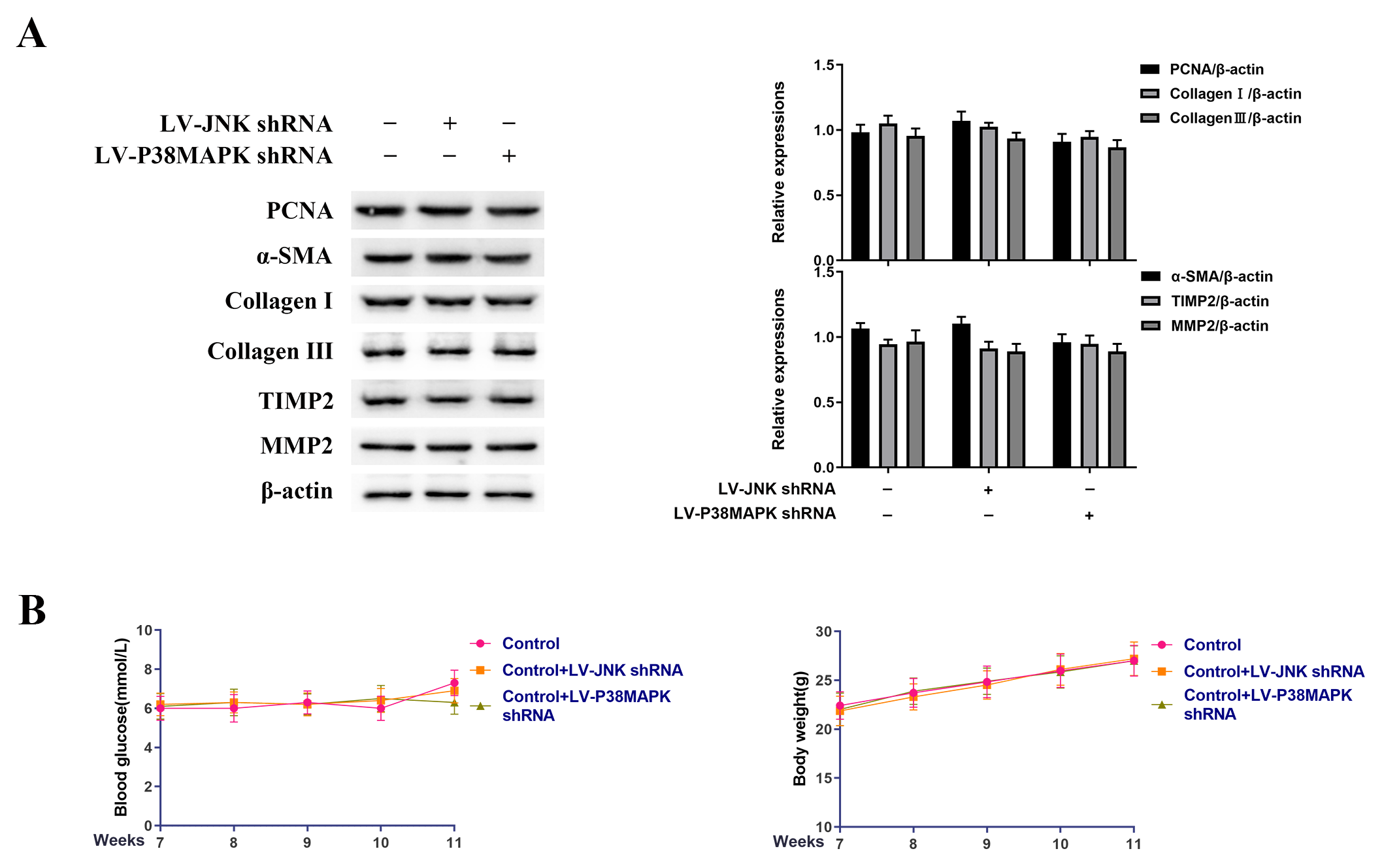

Supplement: Supplementary file 4 [file Image2.TIF]

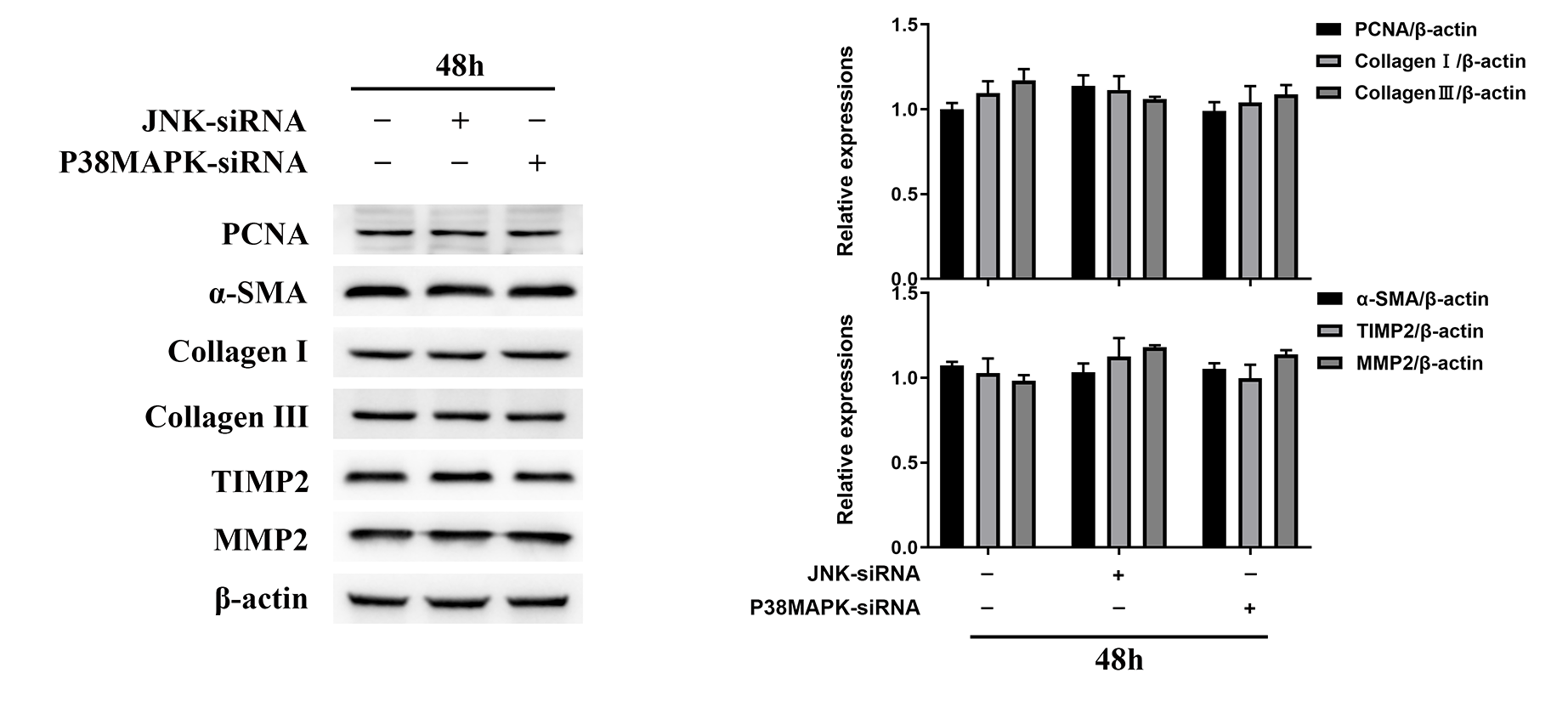

Supplement: Supplementary file 5 [file Image1.TIF]

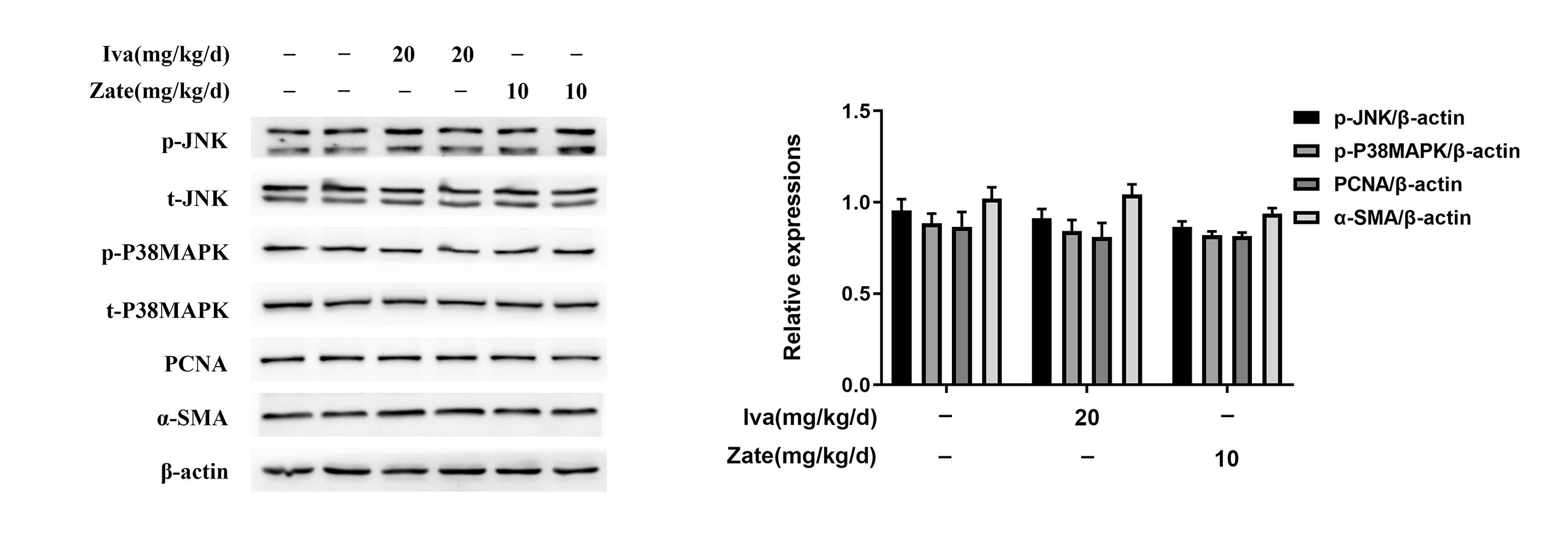

Supplement: Supplementary file 7 [file Image5.TIF]
