# Supplementary material for: Ivabradine Ameliorates Cardiac Diastolic Dysfunction in Diabetic Mice Independent of Heart Rate Reduction
Source: Front Pharmacol. 2021 Jun 22;12:696635. doi: 10.3389/fphar.2021.696635 (PMC8259788; doi:10.3389/fphar.2021.696635)
Supplement: Supplementary file 6 [file DataSheet1.PDF]

SUPPLEMENTARY FIGURE LEGENDS

FIGURE S1 | Effects of the knockdown of JNK or p38 MAPK on fibrogenic proteins *in vitro*. NRCFs were transfected with or without JNK or p38 MAPK siRNA, then the protein expression of PCNA, α-SMA, TIMP2, collagen I, collagen III, and MMP2 was determined by western blotting. Data are presented as mean ± SEM ($n = 3$). *P*-values were calculated using one-way ANOVA with Tukey multiple comparison test.

FIGURE S2 | Effects of the knockdown of JNK or p38 MAPK on fibrogenic proteins, blood glucose concentration, and body mass *in vivo*. **(A)** Expression of PCNA, α-SMA, TIMP2, collagen I, collagen III and MMP2 expression in isolated left ventricular fibroblasts from wild-type mice that did or did not receive a tail vein lentivirus injection 4 weeks earlier. **(B)** Between 7 and 11 weeks following the tail vein injection of lentivirus, the blood glucose concentration and body mass of the wild-type mice were measured. Data in **(A)** ($n = 3$) and **(B)** ($n = 6$) are presented as mean ± SEM. *P*-values were calculated using one-way ANOVA with Tukey multiple comparison test.

FIGURE S3 | Myocardial fibrosis and cardiac diastolic dysfunction (DD) after JNK or p38 MAPK knockdown. **(A)** Masson's trichrome staining of heart tissue in each group (original magnification ×400). Scale bars, 50 µm. **(B)** Echo-Doppler traces for transmitral flow. **(C)** Measurements of E/A, E Dec t, and IVRT made during transmitral pulsed-wave Doppler. **(D)** Hemodynamic parameters (−dP/dt min and the time constant, Tau). Data are presented as mean ± SEM ($n = 6$). *P*-values were calculated using one-way ANOVA with Tukey multiple comparison test.

FIGURE S4 | Effects of ivabradine and zatebradine on the heart rate (HR) and biometric parameters of mice. **(A)** During treatment with ivabradine or zatebradine at the indicated doses for 7–11 weeks, the mean HR of mice was recorded by ECG. **(B and C)** Mice were treated as described in (A) and their blood glucose concentration and body mass were measured. Data are presented as mean ± SEM ($n = 6$). *P*-values were calculated using one-way ANOVA with Tukey multiple comparison test. $^*p < 0.05$, compared to the Control group.

FIGURE S5 | Effects of ivabradine and zatebradine treatment on the activation of JNK and p38 MAPK and the proliferation and activation of CFs *in vivo*. JNK and p38 MAPK phosphorylation and expression of total JNK and p38 MAPK, PCNA and α-SMA in left ventricular fibroblasts isolated from wild-type mice treated with ivabradine or zatebradine at the indicated dose, determined by western blotting. Data are presented as mean ± SEM ($n = 3$). *P*-values were calculated using one-way ANOVA with Tukey multiple comparison test.

FIGURE S6 | Effects of ivabradine and zatebradine on fibrogenic proteins and fibrosis. **(A)** Collagen I, collagen III, TIMP2, and MMP2 protein expression in left ventricular fibroblasts isolated from wild-type mice treated with ivabradine or zatebradine at the indicated doses, determined by western blotting. **(B and C)** Cardiac fibrosis, assessed using Masson's trichrome staining and immunohistochemistry, in the wild-type mice treated as described in (A) (original magnification ×400). Scale bars, 50 μm. Data in **(A)** (n = 3) and **(B–C)** (n = 6) are presented as mean ± SEM. P-values were calculated using one-way ANOVA with Tukey multiple comparison test.
